# Editorial: *In vivo* investigations on neurological disorders: From traditional approaches to forefront technologies

**DOI:** 10.3389/fnins.2022.1052089

**Published:** 2022-10-18

**Authors:** Giulia Curia, Erika Estrada-Camarena, Elias Manjarrez, Hidenobu Mizuno

**Affiliations:** ^1^Department of Biomedical, Metabolic and Neural Sciences, University of Modena and Reggio Emilia, Modena, Italy; ^2^Laboratory of Neuropsychopharmacology, Neuroscience, National Institute of Psychiatry Ramon de la Fuente Muñiz (INPRFM), Mexico City, Mexico; ^3^Institute of Physiology, Benemerita Universidad Autonoma de Puebla, Puebla, Mexico; ^4^International Research Center for Medical Sciences (IRCMS), Kumamoto University, Kumamoto, Japan

**Keywords:** animal models, behavioral tests, bioluminescence, chemogenetics, electroencephalography, magnetic resonance imaging, optogenetics, two-photon imaging

Alterations in neuronal functionality can produce neurological disorders (ND) that may be investigated by *in vitro* or *in vivo* methodologies. *In vivo* investigations performed on animal models of ND provide information about brain activity in pathological and physiological conditions. New technologies recently developed represent a crucial and irreplaceable step in the research on ND. For instance, optogenetics (Huidobro et al., 2017; Kim et al., 2017; Deisseroth, 2021), random noise stimulation (Terney et al., 2008; Herrera-Murillo et al., 2022), and two-photon laser scanning microscopy (Lendvai et al., 2000; Mizuno et al., 2014), complemented with other technologies, such as electrophysiological recordings (Curia et al., 2011) and behavioral tests (Vega-Rivera et al., 2021), may cover several aspects of one pathology.

Electroencephalography (EEG) monitors electrical brain activity in sleepy or awake animals. Analysis of the waveforms can increase knowledge about brain functionality, providing suitable electrical biomarkers to detect a disorder or to follow its progression (Levenstein et al., 2017; Trenado et al., 2019; Girardeau and Lopes-dos-Santos, 2021; Speers and Bilkey, 2021).

Optogenetics is a neurostimulation technique that uses low-intensity light with different waveforms to produce or modulate electrophysiological responses in genetically modified neurons opening promising revolutionary applications in neurological therapeutics in *in vivo* preclinical studies (Biselli et al., 2021; Deisseroth, 2021; Bansal et al., 2022). Recently, opto-non-genetics has been developed, in which neurostimulation with visible light of high-intensity produces inhibition of neuronal firing (Ait Ouares et al., 2019; Ghirga et al., 2020). Interestingly, this last technique could also allow the use of visible light for therapeutic purposes in pathologies related to neuronal hyper-excitability. On the other hand, chemogenetics (Sternson and Roth, 2014; Eisdorfer et al., 2022; Parusel et al., 2022; Singer et al., 2022) is a forefront technique that frequently uses the *in vivo* injection of a viral vector to induce the expression of genetically modified G-protein coupled receptors (GPCR), which are inert for endogenous ligands but specifically activated by “designer drugs.” These expressed receptors are termed DREADDs (Receptors Exclusively Activated by Designer Drugs) (Urban and Roth, 2015; Burnett and Krashes, 2016; Roth, 2016; Smith et al., 2021; Mueller et al., 2022). We can compare it with optogenetics, which employs viral vectors to induce, in excitable cells, the expression of light-activated proteins sensitive to specific types of light (“designer light” at particular wavelengths). In the case of optogenetics, the genetically modified proteins are the opsins (channels or pumps), as the channelrhodopsin-2 (ChR2); in the case of chemogenetics, the genetically modified entities are the DREADS (i.e., the modified GPCRs). Because the use of viral vectors in chemogenetics has the potential to be applied in future clinical trials, then animal research to examine their safety is necessary. Other forms of chemiluminescence include bioluminescence. *In vivo* bioluminescence imaging facilitates the non-invasive visualization of biological processes, such as gene activity in living animals using bioluminescent proteins (Aswendt et al., 2013; Hochgräfe and Mandelkow, 2013).

Two-photon laser scanning microscopy is used for deep tissue imaging in living animals. For instance, the emergence and disappearance of dendritic spines in adult mice (Lendvai et al., 2000) and the dynamic changes in dendrites and axons in developing mice can be observed (Mizuno et al., 2014; Luo et al., 2016; Nakazawa et al., 2018). On the other hand, functional imaging using fluorescent calcium indicators is also possible (Mizuno et al., 2018). Intravital two-photon microscopy should also boost our knowledge of brain circuit formation and circuit changes in ND.

Magnetic resonance imaging (MRI) is a non-invasive multiplanar imaging (image generation) technique, helpful in investigating biological functions with both functional and structural images showing both activity and anatomy (Ikemoto et al., 2022). It is widely used in the neurological field to analyze the presence of ND in humans (Nwosu et al., 2022), and thanks to the relatively recent development of MRI machine for laboratory animals, its use in *in vivo* preclinical investigations has recently grown fast, providing further information about ND (Clemente-Moragón et al., 2022; Ji et al., 2022; Ndode-Ekane et al., 2022).

The behavioral animal models for the study of ND are useful to induce a pathology, mainly after manipulating specific conditions (Belzung and Lemoine, 2011; Kumar et al., 2013; Deguil and Bordet, 2021). Albeit an animal model does not cover all the symptoms of one pathology (validity criteria) (Kumar et al., 2013), their use is a powerful approach to studying the neurobiological bases of ND (McGonigle, 2014; Planchez et al., 2019). Further, the inclusion of behavioral animal models in the study of ND offers the advantage of evaluating the possible factors that may contribute to the development of the problem and the potential treatments to solve it in an integral preparation (Virdee et al., 2012; Phillips et al., 2018; Planchez et al., 2019).

This Research Topic has gathered six original articles and one mini-review from prominent scientists in the field. The collection of papers on this Research Topic provides an up-to-date insight into current knowledge and an overview of different *in vivo* technologies in experimental and clinical ND studies. The content of each of these articles is summarized below.

Stevens et al. examined the optimal features and toxicity levels of a viral vector, the canine adenovirus type 2 (CAV2). In particular, they employed different volumes and viral particle numbers to examine the selective expression, and toxicity levels of a DREADD expressed by CAV2 called hM3Dq, with potential application for chemogenetic modulation of loculs coeruleus noradrenergic (LC-NA) neurons in rats. The authors identified the optimal conditions (low and medium volume with 0.1 × 10^9^ viral particles of CAV2) for the safe and specific transduction of LC neurons with DREADDs technology to study the role of the LC-NA system in health conditions and during specific ND.

Taraschenko et al. found that three different monoclonal antibodies derived from a single encephalitis patient with seizures did not affect motor or anxiety behaviors in mice. Antibody administration and seizures did not alter the expression of astrocytic and microglial markers of inflammation in the hippocampus. However, mice treated with antibodies demonstrated an increased mRNA expression of hippocampal CCL2, a pro-inflammatory chemokine relevant for the persistence of seizures in other seizure models. In particular, higher CCL2 expression correlated with higher seizure burden. The paper by Taraschenko et al. suggests that the development of monoclonal antibodies obviates the need to rely on cerebrospinal fluid supply from affected patients and provides a powerful tool to study the biological effects of antibodies in encephalitis models.

Narcisse et al. contributed to the retinal neurodegeneration study by monitoring the progressive retinal degeneration in the visual cortex in mice with traditional methods to characterize the process and compare it with the Ca^2+^ bioluminescence caption as an index of neuronal activity. The authors used the number of active neurons in the visual cortex and neural activation to measure the progressive deterioration during aging and compared it with the intensity of Ca^2+^-bioluminescence response to visual stimulus. These data are strengthening with the evidence of the correlation between the eyes' electroretinography signal and the retina's thinning (measured by Optical Coherence Tomography) as the index of retinal degeneration progresses. Together this evidence sustains that Ca^2+^ bioluminescence caption imaging constitutes a non-invasive strategy to characterize activities of the visual cortex of retinal degenerative process and constitutes a tool for longitudinal monitoring studies. Also, the authors show the value of bioluminescence over autofluorescence, phototoxicity, and lower resolution electrical methods currently available.

Nakagawa-Tamagawa et al. found that a gain-of-function mutation, I1166T, in Cav1.2 affects neuronal migration and axonal projection during cerebrocortical development. Furthermore, their findings suggested that the Cav1.2 I1166T mutation affects cortical development and callosal projection formation through the Ca^2+^-dependent pathway and β subunit-interaction. These results suggest that Timothy syndrome-like disorder in patients with the Cav1.2 I1166T mutation is associated with abnormal neuronal migration and/or callosal projections.

In their mini-review, Bando et al. summarized the roles of ion channels and transporters in the regulation of electrical properties and Ca^2+^ signaling during neocortical development. They discussed links between abnormal electrical signaling caused by dysfunction of ion channels or transporters and ND. They also discussed the application of optical techniques to address the physiological mechanisms of neocortical development and the pathophysiology of channelopathies.

Atmospheric-pressure gas plasma (APP) is plasma that can be maintained in the surrounding atmosphere without the necessity to apply additional pressure to contain it. Although APP devices were first used for sterilization of contaminated matter (Laroussi, 1996), now they are employed in diverse medical applications. In this Research Topic, Chen et al. evaluated the therapeutic efficacy of this exciting forefront technique. In particular, they applied intermittent inhalation of gas plasma (APP jet) in a rat ischemic stroke model. These authors found that post-stroke treatment with this APP jet intervention could reduce the ischemic lesion progression and decrease cerebral infarction volume, which might provide a new promising technology for ischemic stroke treatment (Kuriakose and Xiao, 2020).

Rao et al. found that dendritic patterning and synapse formation are impaired in RasGAP-suppressed neurons in the cerebral cortex. The findings provided insights into the pathophysiology of brain disease due to dysfunction of RasGAPs, such as the causative gene of neurofibromatosis type I. The results suggested that dendritic and synaptic development changes could be associated with the cognitive disabilities seen in patients with neurofibromatosis type I.

## Conclusions

In this Research Topic, we describe the peculiar features of several traditional and forefront technologies and present some of their applications, demonstrating the importance of preclinical research in neuroscience and showing that the replacement of laboratory animals is not always possible.

## Author contributions

GC, EE-C, EM, and HM contributed to the article and approved the final version for publication.

## Conflict of interest

The authors declare that the research was conducted in the absence of any commercial or financial relationships that could be construed as a potential conflict of interest.

## Publisher's note

All claims expressed in this article are solely those of the authors and do not necessarily represent those of their affiliated organizations, or those of the publisher, the editors and the reviewers. Any product that may be evaluated in this article, or claim that may be made by its manufacturer, is not guaranteed or endorsed by the publisher.
